# Factors leading to dyspepsia in renal transplant recipients

**DOI:** 10.11604/pamj.2017.28.120.12767

**Published:** 2017-10-06

**Authors:** Aisha Nazeer, Ayesha Aslam Rai, Nasir Hassan Luck

**Affiliations:** 1 Department of Hepatogastroenterology, Sindh Institute of Urology and Transplantation, Karachi

**Keywords:** Renal transplantation, dyspepsia, tacrolimus

## Abstract

**Introduction:**

Renal transplantation is the definitive treatment for end stage renal disease. Patients subjected to transplantation require lifelong immunosuppression and are prone to several gastrointestinal disorders. Dyspepsia is a common disorder in these patients. The objective of this study was to determine factors leading to dyspepsia in renal (kidney) transplant recipients.

**Methods:**

It was a cross sectional study conducted at department of hepatogastroenterology and transplant sciences, SIUT Karachi, from 1-6-15 to 1-12-15 for six months. All renal transplanted patients having dyspeptic symptoms for more than 6 weeks. EGD was performed, biopsy specimens obtained from antrum and duodenum, these were sent for histopathological examination. Frequency and percentages were obtained for categorical variables, mean ± SD was calculated for continuous variables. Chi square test was used for categorical variable and student t-test for continuous variables.

**Results:**

Ninety patients were included in the study out of which 64 (71.1%) were males, mean age was 35.82 ± 10.04 years (range: 18-65 years). Gastritis (non *H.pylori* associated) in 78 (78.6%), duodenitis in 35 (38.9%) and *H. pylori* infection in 29 (32.2%), renal transplant recipients. Most of the patients belonged to Sindhi ethnicity, 27 (30%), followed by Punjabi. Hypertension was the most common co-morbid condition in our patients found in 29 (32.2%), while most of them don't have any co morbid condition. Duodenitis was found to be associated with tacrolimus use (p = 0.037).

**Conclusion:**

Gastritis is the most common factor accountable for this symptoms, followed by duodenitis and *H. Pylori*. Patients taking tacrolimus as immunosuppressant are more prone to develop duodenitis.

## Introduction

Dyspepsia and gastroesophageal reflux disease (GERD) are the most common diseases encountered by the gastroenterologist, its prevalence in general population is about 10-20% in the Western world and lower in Asia. Six percent of the patients have heartburn; around 38% had dyspepsia while regurgitation was reported in 16% [1, 2]. GERD develops when increased acidic gastric secretion refluxes into the esophagus [3, 4]. Gastrointestinal (GI) symptoms are commonly experienced in solid organ transplant recipients and can effects any part of the GI tract [5]. After three years of renal transplant, about 23% of the patients have GERD, reflux esophagitis (RE) and dyspepsia in 20%, 5% and 6%, respectively [6]. Renal transplantation, chronic renal disease, immunosuppressive therapy, *H. pylori* infection, female gender, pre transplant upper gastrointestinal disease, older age, obesity, Caucasian and African-American race, are considered as potential risk factors for the development of GERD and dyspepsia [6–9]. Neri et al concluded that female gender (39.9%), hypertension (22.5%) and renal failure secondary to type I DM (12.1%) have increased risk of dyspepsia [6]. Raised creatinine is one of the risk factor of dyspepsia in post renal transplant era [7, 8]. In normal population increased age and smoking are risk factors of dyspepsia [9]. Darji et al found that 50% of patients had considerable improvement in dyspeptic symptoms after substituting mycophenolate mofetil (MMF) with its enteric coated formulation [10]. Besides Webster et al concluded that in comparison to cyclosporine, the use of tacrolimus was associated with more risk of dyspepsia, post-transplant diabetes mellitus (PTDM), diarrhea, tremor and headache [11] Ozgur et al inducted 54 renal transplanted patients with dyspepsia out of which *H. pylori* infection was seen in 70%, gastritis in 65%, duodenitis in 13% and peptic ulcer in 4% [8]. Khidmat et al found duodenal ulcer, erosive gastritis and *H. pylori* infection in 8%, 20.5% and 40% respectively in post renal transplant [12]. Neri et al proposed that renal transplanted patients having GERD, dyspepsia or reflux esophagitis are at increased risk of graft failure and death [6]. Kim et al reported severe complications in around 10% resulting in graft loss and death [5]. The rationale of this study is to determine factors leading to dyspepsia in renal transplant recipients, as no local study have been done so far for evaluation of dyspepsia in these patients. Evaluation of factors leading to dyspepsia, its early diagnosis and prompt treatment is crucial in these patients as it can lead to graft failure and increased morbidity and mortality.

## Methods

The study was descriptive cross sectional study, conducted in the department of Hepatogastroenterology, Sindh Institute of Urology and Transplantation (SIUT), Karachi from 1^st^ June 2015 to 30^th^ November 2015. Those patients who had dyspepsia^3^ for at least 6 weeks after 6 months of renal transplantation of both genders and aged 18 and above were included in the study. Following patients were excluded: those who had taken proton pump inhibitors at least two weeks before EGD, had congestive heart failure (i.e: ejection fraction is less than 40% on echocardiography), bleeding disorder: thrombocytopenia or coagulopathy (Platelets < 50000/μL or International normalized ratio (INR) > 1.5, hemodynamically unstable (B.P < 70/40 mmHg). Eight factors which lead to dyspepsia were taken into consideration in this study, factors were gender, hypertension, diabetes mellitus, obesity, immunosuppressive drugs, *H.pylori* gastritis, non *H.pylori* gastritis and duodenitis. Sample size was calculated by using open epi calculator prevalence of duodenitis with dyspeptic symptoms was found to be 13% [8] in renal transplant recipients, d = 7%, confidence interval = 95%, estimated sample size was at least n = 89. All the cases were subjected to endoscopic evaluation. Venous blood sample collected for laboratory parameters; urea, creatinine, complete blood count and prothrombin time. Esophagogastroduodenoscopy (EGD) was done in all enrolled patients as per indication, performed by researcher under supervision of supervisor using video endoscope (Olympus GIF-XP180). Two biopsies were taken from antrum and duodenum for histopathological examination and were sent to the histopathology department at SIUT, to rule out gastritis, duodenitis or *H. pylori* infection. Data was analyzed by using statistical software SPSS-20.0. Mean and standard deviation (SD) was calculated for continuous variables (age, BMI, duration of symptoms). Frequencies and percentages were evaluated for categorical variables (Gender, co-morbids ie DM and HTN, addictions, *H.pylori*gastritis, duodenitis, gastritis, immunosuppressants which includes steroids, azathioprine, everolimus, tacrolimus, cyclosporine, mycophenolate mofetil and obesity). Stratification with respect to age, addiction, ethnicity, duration since renal transplant and duration of symptoms was done. Post stratification Chi square test was applied. A p-value ≤ 0.05 was taken as significant.

## Results

Total 90 renal transplant recipients were enrolled in this study. Most of the patients were male i.e 64 (71.1%) (Table 1). Mean age was 35.82 ± 10.04 years (Range: 18-65 years) (Table 2). Most of the patients belonged to Sindhi ethnicity, 27(30%) followed by Punjabi 21 (23.3%) (Table 3). Almost half of the patients had no known co- morbid 47 (52.2%). Hypertension, DM and hepatitis B was observed in 29 (32.2%), 4 (4.4%) and 4 (4.4%) respectively (Table 4). Upper GI symptoms before transplant were also uncommon in these patients, only around one-fourth of subjects had history of dyspeptic symptoms prior to transplant i.e: 23 (25.6%). Sixty nine patients (76.7%) had no history of substance abuse while tobacco smoking was observed in 12 (13.3%) subjects followed by alcoholism in 6 (6.7%) (Table 1). In the study subjects, urea was ranged from 17-230 mg/dL with mean of 58.34 ± 39.30 mg/dl, while creatinine ranged from 0.72-6.08 mg/dL, with mean of 2.11 ± 1.11 mg/dL (Table 2). Renal transplant patients were receiving multiple immunosuppressant, all of them were on steroids (prednisolone) along with other agents. Azathioprine was used in 54 (60%), followed by cyclosporine in 35 (38.9%), tacrolimus in 27 (30%), MMF in 26 (28.9%) and everolimus in 8 (8.9%) patients. Gastritis (non *H. pylori* infection) seen in 78 (86.7%), duodenitis in 35 (38.9%) and *H. pylori* infection in 29 (32.2%) recipients as cause of dyspepsia (Table 1). No endoscopy related complication was observed in any patient. Analysis of factors leading to dyspepsia, revealed statistically significant association in patients having duodenitis on tacrolimus (p = 0.037) (Table 5), however, one tailed p-value was significant in patients having gastritis and BMI of more than 18.92 (p = 0.053). No significant association was found with other immunosuppressive agents in patients having *H. Pylor*i, non *H. Pylori* gastritis, duodenitis or female gender.

**Table 1:** Demographic and clinical characteristics of study population (n=90)

Factors	Number of patients	Percentage %
Female	26	29
Male	64	71
HTN	29	32.2
DM	4	4.4
Obesity	2	2.2
H. *P*ylori gastritis	29	32.2
Non H. Pylori gastritis	78	86.7
Duodenitis	35	38.9
Steroid	90	100
Azathioprine	54	60
MMF	26	28.9
Everolimus	8	8.9
Tacrolimus	27	30
Cyclosporin	35	38.9
**Substance abuse**		
None	69	76.7
Tobacco	12	13.3
Alcohol	6	6.7
Betel nuts	2	2.2
Tobacco and Alcohol	2	1.1

**Table 2:** Descriptive statistics of continuous variables (n=90)

Variables	Mean	Range	± SD
Age (Years)	35.82	18-65	10.04
Creatinine (mg/dL)	2.11	0.72-6.08	1.11
BMI (Kg/m^2^)	19.67	12.94-32.06	3.65
Urea (mg/dL)	58.34	17-230	39.3
Duration of symptoms in weeks	11.49	20-7	3.47
Duration since transplant in months	81.48	328-8	54.78

**Table 3:** Ethnicity of study population (n=90)

Ethnicity	Number	Frequency
Sindhi	27	30
Punjabi	21	23.3
Balochi	7	7.8
Pathan	7	7.8
Urdu	17	18.9
Siraiki	6	6.7
Memon	1	1.1
Kashmiris	1	1.1
Others	3	3.3

**Table 4:** Co morbid of study Population (n=90)

Co-morbid conditions	Number	Percentage
DM*	4	4.4
HTN*	29	32.2
Hepatitis B	4	4.4
Hepatitis C	3	3.3
Depression	2	2.2
Others	1	1.1
None	47	52.2

**Table 5:** Analysis of factors leading to dyspepsia: duodenitis (n=90)

Variables	Duodenitis	Two tailed p – Value
**Creatinine** mg/dL < 1.745	15	0.387
>1.745	30	
**Urea** < 45 mg/dL	20	0.666
>45 mg/dL	15	
**Age**	18	1.0
<35 years		
<35 years	17	
**Tacrolimus**	6	0.037
**Duration of Symptoms**		1.000
<12 weeks	17	
>12 weeks	18	
**Duration since Transplant**		0.611
< 74.5 months	14	
>74.5 months	12	
**Addictions**		0.702
None	26	
Tobacco	4	
Alcohol	3	
Betel nuts	1	
Tobacco and Alcohol	1	
**Ethnicity**		0.793
Sindhi	11	
Punjabi	10	
Balochi	2	
Pathan	3	
Urdu	6	
Saraiki	1	
Memon	0	
Kashmiri	0	
Others	2	

## Discussion

Renal transplantation is the definitive management for patients suffering from of end stage renal disease (ESRD) [13]. Gastrointestinal disorders in this population are more common as compared to general population, which includes oral ulcers, esophagitis, peptic ulcer disease, diarrhea and dyspepsia [14]. Post renal transplant dyspepsia not only worsens the quality of life, but is also associated with increased risk of graft failure, resulting in high morbidity and mortality [6]. Among the demographic characteristics, the gender distribution in our study was similar in comparison with other studies. Our study reported that around 70% of the patients were male, consistent with multiple other studies in which around 60-70% of the patients were male [6–8, 15–17]. There is no statistical significance seen in our study as well as in previous studies. Mean age of our study group was 35.82 ± 10.04 years, which was lower than that seen in other regions of world. Around 35% of the patients, who underwent renal transplant in USA belonged to the age group of 45-60 years [6], similar characteristics were seen by Ozgur et al [8] and Savas [15]. Our contrasting results could possibly be explained by the fact that we belong to a developing country in the stone belt and are unable to identify early detection of renal diseases and implement strategies to delay the progression of renal failure. These factors probably play an important role in the development of dyspepsia in renal transplanted patients and ESRD at younger age in our country [13]. Most of the patients in our study had normal BMI with mean of 19.67 ± 3.65, which is in contrast to the study done at USA on renal transplanted subjects, [6] they had mean BMI of 44 ± 12.5 kg/m^2^. This huge difference of BMI is likely because our patients belonged to a third world country with limited resources. Our transplanted patients follow stringent criteria of weight maintenance, as advised by nutritionist and most of them are compliant.

In our study, no statistically significant association of dyspepsia was found with ethnicity and obesity, which was different from the results of Neri et al [6] that found ethnicity as important factor and concluded that whites, Hispanic and African-Americans who are obese and have greater BMI were more prone to develop GERD and dyspepsia. Most of our patients in our study underwent renal transplant were non-diabetics, hypertension was the most common co morbid condition, this is probably because we live in the region of stone belt and likely this would be the common cause of ESRD in our transplanted patients [13]. Neri et al also have same results, found HTN to be the most common cause of renal failure, followed by DM [6]. In our region most of the diabetic patients are unable to avail this treatment option because of multiple co morbidities and complications related with DM. In this study about one-fourth of subjects that is, 23 (25.6%), had dyspeptic symptoms before transplant; however no significance of this observation can be elicited because there was no control group. It was seen by Neri et al that those patients who had upper GI symptoms, prior to transplant, were more prone to experience it post-transplant as well [6]. Mean urea and creatinine in our study was 58.34 ± 39.3 mg/dL and 2.11 ± 1.1 mg/dL respectively, this was higher than that of Saudi Arabia's population, have mean creatinine of 1.6 ± 1.1 mg/dL, but this had no association with factors leading to dyspepsia [7]. Deranged urea and creatinine, has been considered as one of the known cause of dyspepsia in these patients [6].

The most common factor leading to dyspepsia in this study was gastritis seen in 78 (86.7%), followed by duodenitis and *H. Pylori* infection. A study done at Istanbul also finds gastritis as the most common finding on endoscopy, seen in 55 (85.9%) patients [15]. Abdur Rehman and Al Qurain's observation was contrasting and found that GERD was more common in renal transplanted patients in comparison with gastritis [7]. In our study frequency of *H. Pylori* was less as compare to the other studies. Thirty two percent of our patients were found to have *H. pylori* infection in comparison to Ozgur et al and Hurby's, who reported it this infection in 70 and 62% respectively [8, 17]. Another study done at the same institute as ours (SIUT) reported *H. pylori* infection in 11 patients out of 119 examined gastric biopsies, showing decrease prevalence of this infection in our renal transplanted population [18]. A possible explanation of the differences of these results is yet to be known. This study has highlighted the statistical significance of tacrolimus with duodenitis. A case report has been published by Suzuki et al he found severe duodenitis along with graft dysfunction following renal transplantation, the patient had various immunosuppressants to avoid rejection including tacrolimus [19]. To the best of our knowledge, previously no such association has been reported in a study. Although this study has tried to identify various factors leading to dyspepsia, there is still need for further research work in this regard. It was a single center study, with small sample size. Therefore, further studies are needed to assess other factors leading to dyspepsia in renal transplant recipients.

## Conclusion

The common cause of dyspepsia in our renal transplanted patients was gastritis (non *H. pylori* associated) followed by duodenitis and *H. pylori* infection. Use of immunosuppression tacrolimus was associated with duodenitis.

### What is known about this topic

- Dyspepsia is common in uremic patients;
- Renal transplant recipients are prone to have dyspepsia because of immunosuppressive agents.

### What this study adds

- Most common cause of dyspepsia is non *H.pylori* gastritis;
- Duodenitis is associated with the use of tacrolimus as immunosuppressant.

## Competing interests

The authors declare no competing interests.
